# The Rapid Growth of Burkitt Lymphoma Causing Partial Small Bowel Obstruction

**DOI:** 10.7759/cureus.56227

**Published:** 2024-03-15

**Authors:** Zackary D Anderson, Alex Ashkin, Leslie Raymond

**Affiliations:** 1 Graduate Medical Education/Internal Medicine, Naples Community Hospital, Naples, USA

**Keywords:** non-hodgkin’s lymphoma, small bowel obstruction, tumor lysis syndrome, endemic burkitt lymphoma, sporadic burkitt lymphoma

## Abstract

Burkitt lymphoma (BL) is a neoplasm of the lymphoid tissue and one of the most prevalent malignancies worldwide. Classically, these patients present with unregulated B-cell differentiation causing fever, chills, night sweats, and weight loss. Although more common in children, in sporadic Burkitt lymphoma, symptoms often can be present in the abdomen. These patients also additionally report nausea, vomiting, and abdominal distention, which in rare instances can cause small bowel obstruction (SBO). Early detection and the initiation of chemotherapy remain highly effective in providing adequate care. This provides better outcomes and prevents surgical management.

## Introduction

Non-Hodgkin’s lymphoma (NHL) is a neoplasm of the lymphoid tissue and one of the most prevalent malignancies worldwide [1]. A variant of NHL is Burkitt lymphoma (BL), which often develops from germinal or post-germinal B-cells [1]. It accounts for 1%-5% of NHL cases with a higher prevalence in males of Caucasian descent. The subtypes of BL include sporadic, endemic, and immunodeficiency-associated. The sporadic form is far more common in pediatric populations representing 30% of pediatric lymphomas, with less than 1% of all NHL in the adult population being sporadic [2]. This form is typically associated with Epstein-Barr virus (EBV) and most commonly involves the abdomen [2]. Typically, lymphomas present with symptoms related to unregulated B-cell differentiation causing fever, chills, night sweats, and weight loss. However, in sporadic Burkitt lymphoma, symptoms often additionally relate to the location of the mass. These patients also additionally report nausea, vomiting, and abdominal distention. With an average age of onset being six years old, most cases report complications in the pediatric population. The prevalence of these complications is something that is not well documented in the medical literature. It can be treated with systemic chemotherapy, and the early initiation of treatment can provide shrinkage of the tumor. This leads to the resolution of abdominal symptoms and prevents unnecessary surgical risk. Here, we represent a rare case of rapidly progressing Burkitt lymphoma leading to partial small bowel obstruction (SBO) in an adult.

## Case presentation

Here, we have a 63-year-old male with a past medical history of hypertension, who presented with concerning features of bloating and constipation over a two-week time period. The patient had a colonoscopy two months prior that showed no evidence of polyps or any abnormalities notable for concern. The patient had no significant risk factors including environmental exposures, a history of malignancy, smoking, or excessive alcohol use. He did not have a family history of cancer. On arrival, the patient had a respiration rate of 18, with an initial blood pressure of 115/63 mmHg and a heart rate of 76 beats per minute (bpm). The patient was afebrile and in no acute distress. On a physical examination, the patient was found to have a normal sinus rhythm, and the lungs were clear to auscultation bilaterally. His abdomen was found to be markedly distended, with no fluid wave present. The patient had hypoactive bowel sounds and had tenderness to palpation in all four quadrants. On initial laboratory studies, the patient was found to have a complete blood count showing a hemoglobin of 13.6 g/dL and hematocrit of 40.8%, with neutrophil-predominant leukocytosis of 11.6%. On the complete metabolic panel, the patient was found to have electrolytes within normal limits, albumin of 3.2 g/dL, alkaline phosphatase of 198 U/L, aspartate aminotransferase (AST) of 74 U/L, alanine aminotransferase (ALT) of 79 U/L, and lipase and amylase within normal limits.

**Figure 1:**
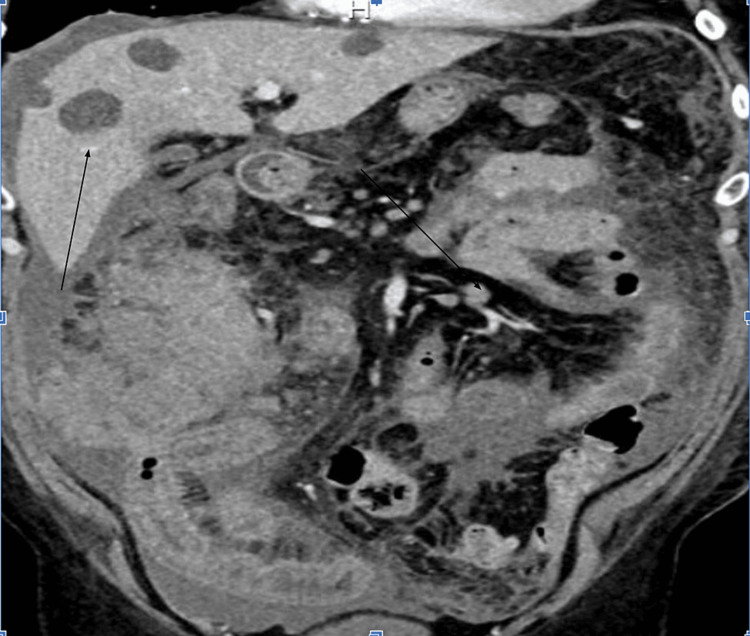
Hypodense liver metastasis with omental caking

The initial CT of the abdomen and pelvis showed a large 14.4 × 12 × 14 cm irregular thickening with a heterogeneous mass involving the distal rectum, cecum, and ascending colon concerning neoplasm with hypodense lesions throughout the liver and omental caking (Figure 1). The patient was admitted in the setting of metastatic disease of unknown origin, with anticipation and plans for a biopsy to further establish a diagnosis. During the admission, the patient underwent an interventional radiology-guided biopsy and remained admitted awaiting pathology results. The patient was maintained on intravenous (IV) fluid hydration and antiemetics but awaited curative treatment until pathology resulted. Over the next couple of days, the patient developed worsening abdominal pain and increased distention and had yet to have a bowel movement.

Due to concern for underlying obstruction, repeat imaging was performed, which revealed a worsened thickening of the ileum, with growing concerns for bowel obstruction. Early consultation with general surgery was made with the hopes that early aggressive conservative management would avoid the need for surgical intervention. The surgical recommendations were consistent with gathering further pathology as this presentation was consistent with a rapidly growing malignancy. The patient was placed on fluids and remained nil per os. Fortunately, he showed significant improvement, and we were able to avoid the need for surgical intervention or nasogastric tube placement. Shortly thereafter, the pathology results from a prior biopsy revealed Burkitt lymphoma (c-Myc+ and B-cell lymphoma 2 {Bcl-2-}). The patient was started on modified etoposide, rituximab, prednisone, vincristine, sulfate (Oncovin), cyclophosphamide, doxorubicin, and rituximab (EPOCH-R chemotherapy regimen). The initiation of this chemotherapy avoided any surgical interventions as the standard of care is chemotherapy. Clinically, the patient was intervened in a timely manner, which gave the chemotherapy adequate time to allow for a reduction in symptoms. The patient completed multiple rounds of chemotherapy and followed up in the outpatient setting with oncology. The patient is now in full remission.

## Discussion

With the majority of Burkitt lymphoma cases presenting in children and young adults, Burkitt lymphoma is often overlooked in older adults. A general suspicion that arises most often for lymphoma is one centered around fever, chills, and night sweats. However, sporadic Burkitt lymphoma presents with abdominal symptoms and even in rarer instances those related to underlying small bowel obstruction. The specifics regarding statistical data are unavailable, but key instances are documented in the literature. The key importance of early diagnosis is crucial and often starts with a thorough medical history.

Generally, in adults, small bowel obstruction is one that is associated with adhesions, strictures, and localized or metastatic malignancies causing the onset of symptoms. In the case of our patient, he had no prior surgical history and did not have a history of inflammatory bowel disease. Additionally, he had a recent colonoscopy with no evidence of even polyps present. This lack of evidence on colonoscopy has been proven favorable to rule out colorectal malignancies.

A retrospective study of 804 patients with later diagnosed colorectal cancer showed patients with known masses to have a median doubling time of 211 days [3]. In contrast, Burkitt lymphoma is highly aggressive with an average doubling time of 25 hours [4].

In conjunction with a complete history, specific laboratory studies can aid in a lymphoma etiology of obstruction. These laboratory studies include nonspecific markers such as uric acid, lactic dehydrogenase (LDH), hyperkalemia, hyperphosphatemia, and hypocalcemia [5]. Burkitt lymphoma has a 14% chance of causing tumor lysis syndrome even prior to the initiation of chemotherapy treatment. It can be useful to evaluate these laboratory studies as these hallmark values can provide further evidence of a lymphoma diagnosis. In our particular case, tumor lysis laboratory studies were evident in the earliest stages of hospitalization.

The acute management of SBO is dependent on early diagnosis and treatment, and this often includes imaging studies. The gold standard is CT of the abdomen with IV contrast [6]. Although CT plays a crucial role, it does not display key characteristics that could differentiate masses further. A key imaging study shown to be helpful in providing clues of the mass’s origin is an abdominal ultrasound. On ultrasound of the abdomen, features of BL often appear as high-flow areas with circumcised, uniformly hypoechoic masses correlating to diffusely infiltrating lymphoma cells in intestinal submucosal layers [7].

In most instances, treatment options for small bowel obstruction are dependent on what is causing the obstruction. Generally, masses are often recommended for general surgery consultation as resection is the treatment of choice. Early surgical intervention in relation to tumors has remained standard practice as it leads to decreased episodes of bowel necrosis and further complications.

Surgery remains an option for BL in localized disease but generally is not recommended if disseminated or metastatic disease is present as was the case in our patient [5]. The overall risk stratification is not one that has been fully studied in adults. A study performed in 1982 showed that adolescents who underwent surgery and chemotherapy had a significant cure rate. Although that may be the case, this came with complications like those of other elective procedures. Of the 92 cases, 6.5% had postsurgical infections, with an 11% incidence rate of intra-abdominal abscesses [8]. Although not a significant risk compared to other surgeries, not exposing a patient to surgery and providing gold standard treatment should be the main priority.

This can be achieved through early detection and high levels of suspicion. This detection is achieved through confirmation via biopsy. The microscopic analysis for BL includes “starry skies” or sheets of intermediate-sized lymphocytes with dispersed histiocytes. *c-MYC* gene translocation on chromosome 8 is a hallmark of the disease, mostly of the t(8;14)(q24;q32) genotype [9,10]. Flow cytometry for B-cell lymphoma consists of a kappa light chain with the expression of B-cell markers, which are useful [5].

With an early diagnosis, treatment that revolves around chemotherapy can be initiated. In adult populations who receive a regimen of EPOCH-R, studies have shown a complete remission rate of 79% and a mean overall survival of 64%. With such an effective response, it provides further importance and awareness of this clinical vignette to the literature.

## Conclusions

Although primarily a pediatric tumor, Burkitt lymphoma in adults is one worth considering when patients present with bowel obstruction related to an underlying mass. This disease process is worth considering as early detection and the initiation of chemotherapy remain highly effective in providing adequate care. This overall provides better patient outcomes, is a guideline therapy, and prevents unnecessary surgical intervention.
